# Comparison of the World Health Organisation and International Consensus Classification of haematolymphoid tumours

**DOI:** 10.4102/jcmsa.v2i1.75

**Published:** 2024-09-04

**Authors:** Sumaiya Cassim, Erica-Mari Nell, Ethan J. Gantana, Ernest M. Musekwa, Robert K. Lohlun, Ibtisam Abdullah, Zivanai C. Chapanduka

**Affiliations:** 1 Division of Haematology, Department of Pathology, Faculty of Medicine and Health Sciences, Stellenbosch University, Cape Town, South Africa; 2 Division of Haematology, Department of Pathology, National Health Laboratory Service, Tygerberg Hospital, Cape Town, South Africa; 3 Division of Haematology, Department of Pathology, Health New Zealand Te Whatu Ora, Wellington, New Zealand

**Keywords:** WHO-HAEM4R, WHO-HAEM5, ICC, myeloid tumours, lymphoid tumours

## Abstract

**Background:**

The World Health Organization has updated its classification of haematolymphoid tumours to the new 5th edition. The International Consensus Classification is another group that has developed a separate classification system with similar but conflicting approaches. This led to a debate among haematologists about which classification system is more appropriate for clinical use. This article examines and compares these contrasting and concurring classifications, and provides insight into the utility of each system.

**Methods:**

Journal articles pertaining to WHO-HAEM4R, WHO-HAEM5 and ICC were searched for using the MEDLINE database, from November 2022 to November 2023. Original research articles and reviews were selected to compile this review.

**Results:**

The classification systems share many similarities with minor name changes and subgrouping. There are few instances where diagnostic grouping differs between the classifications, which could have clinical implications regarding treatment and enrolment in clinical trials.

**Conclusion:**

There is universal agreement about the need for objective criteria for the classification of haematolymphoid tumours. As a result, there have been considerable strides in the classification with regard to morphologic, immunophenotypic, molecular and cytogenetic characterisation. It is reassuring to have a stepwise approach to diagnosis, allowing developing countries to make appropriate diagnoses.

**Contribution:**

Harmonisation is needed for a universal diagnostic system for the benefit of the patient. Both classification systems have merit and either could be adopted by individual institutions at present. The authors appeal for interim advocacy measures to assure access to the specific diagnostic investigations in poorly resourced societies, pending more permanent and sustainable access.

## Introduction

The World Health Organization (WHO) has been instrumental in developing disease classification systems which enable health professionals to reach consensus on the diagnosis and treatment of patients. With the update of the latest WHO classification of haematolymphoid tumours from the revised 4th edition (WHO-HAEM4R) to the new 5th edition (WHO-HAEM5), another group has developed a separate classification, the International Consensus Classification (ICC), which has similar but conflicting approaches to classifying myeloid and lymphoid neoplasms.^1,2,3^ The ICC was developed by clinical advisory committees composed of several expert pathologists, haematologists, oncologists and geneticists, many of whom were authors of the previous WHO editions. This led to a debate among haematologists about which classification system is more appropriate for use in clinical diagnosis and trials. This article examines and compares the contrasting and concurring classifications of the WHO-HAEM5 and the ICC, and provides insight into the utility of each classification system.

## Method

This literature search was conducted from November 2022 to November 2023. All seven authors were involved in the selection process. Journal articles pertaining to the WHO-HAEM4R, WHO-HAEM5 and ICC were searched for using the MEDLINE database. Approximately 37 000 publications were yielded. Current original research articles and reviews pertaining to haematolymphoid tumours were selected from this reference list, which amounted to 144. Fifteen citations of these publications were also selected. A total of 159 publications were then used to compile this review. This was then significantly edited to comply with journal requirements resulting in a final reference list of 59 publications. A comparison of the classification systems was also collated in table format (Online Appendix 1).

## Myeloid neoplasms

### Myeloproliferative neoplasms

In the WHO-HAEM5, chronic myeloid leukaemia in accelerated phase (CML-AP) is omitted because overall survival (OS) has improved in chronic myeloid leukaemia in chronic phase (CML-CP) and *de novo* CML-AP as a result of tyrosine kinase inhibitor (TKI) therapy.^4,5^ Notably, there is minimal OS benefit of TKI therapy in CML-AP that has evolved from CML-CP.^5^ To accommodate this, the WHO-HAEM5 introduces high-risk features of CML-CP, which include the criteria for CML-AP, as retained and defined by the ICC. Clinical management of CML-CP with high-risk features (WHO-HAEM5) would therefore be the same as CML-AP (ICC), but the differing terminology may become problematic for enrolment in clinical trials. The ICC elects the threshold of > 5% lymphoblasts in the peripheral blood (PB) or bone marrow (BM) to signify consideration for a lymphoblastic crisis, while the WHO-HAEM5 suggests that there is currently insufficient evidence to specify a suitable threshold.

There is consensus between the classifications for *BCR::ABL1*-negative myeloproliferative neoplasms (MPNs) other than polycythaemia vera (PV). The WHO-HAEM5 omits the red cell mass criterion for the diagnosis of PV as it is uncommonly performed. Both classifications allow diagnosis of PV without BM morphology if other specified criteria are fulfilled. Importantly, the ICC emphasises the use of highly sensitive single-target molecular assays with a minimum sensitivity of 1% to detect low variant allelic fractions (VAF). This could be challenging in limited resource settings.

Regarding chronic eosinophilic leukaemia (CEL), the qualifier not otherwise specified (NOS) is omitted from the WHO-HAEM5 as it is considered a well-characterised disease. Not otherwise specified is retained by the ICC. Both classifications include abnormal BM morphology (dysplasia) as a criterion, which allows better differentiation from related hypereosinophilic entities. The ICC requires relative eosinophilia in addition to absolute eosinophilia for diagnosis of CEL, NOS, improving the specificity of this criterion. The WHO-HAEM5 includes clonality as a required criterion. This change makes the diagnosis of CEL challenging, especially in the context of clonal haematopoiesis of indeterminate potential (CHIP) and in settings with limited access to next-generation sequencing (NGS).

Myeloproliferative neoplasm, unclassifiable, as retained in the ICC, is renamed to MPN, NOS in the WHO-HAEM5 to omit the paradoxical qualifier unclassifiable.

Juvenile myelomonocytic leukaemia (JMML), previously categorised in the WHO-HAEM4R as a myelodysplastic and myeloproliferative neoplasm (MDS/MPN), is categorised in the WHO-HAEM5 as MPNs and in the ICC as paediatric and/or germline mutation-associated disorders. Both classifications omit monosomy 7 as a criterion, highlighting the lack of myelodysplastic features in this disease.

### Myelodysplastic and myeloproliferative neoplasms

The threshold for absolute monocytosis in chronic myelomonocytic leukaemia (CMML) is lowered to 0.5 × 10^9^/L in both classifications, which allows for the inclusion of cases previously referred to as oligomonocytic CMML (OM-CMML).^6^ However, monocytosis of 0.5 to < 1.0 requires proven clonality and evidence of dysplasia in at least one lineage. Development of an *NPM1* mutation in CMML qualifies it as acute myeloid leukaemia (AML) regardless of the blast count, according to the WHO-HAEM5, whereas the ICC requires ≥ 10% blasts.

Nevertheless, mutated *NPM1* appears to herald a particularly aggressive course and deserves AML-like management.^7^ Both classifications acknowledge the role of immunophenotyping in differentiating CMML from other causes of monocytosis without clonality. However, the specificity and sensitivity of such assays have been challenged by recent studies.^8^

Chronic myelomonocytic leukaemia-0 is omitted from both classifications and reverts to a two-tier system of CMML-1 and CMML-2 because CMML-0 has limited prognostic significance.^9^ The subtypes of CMML, dysplastic type (MD-CMML; WBC < 13 × 10^9^/L) and proliferative type (MP-CMML; WBC ≥ 13 × 10^9^/L), are officially recognised in both classifications based on evidence of poorer outcomes for MP-CMML.^10^ A new precursor entity, clonal monocytosis of undetermined significance (CMUS), characterised by monocytosis > 10% and the presence of myeloid neoplasm-associated mutations at a VAF of ≥ 2% in the absence of morphologic findings of CMML, is recognised by the ICC in the MDS/MPN category. Recategorisation of this precursory condition in the clonal haematopoiesis section seems more appropriate.

Atypical CML (aCML) is renamed MDS/MPN with neutrophilia in the WHO-HAEM5 to avoid confusion with *BCR::ABL1*-positive CML and to emphasise the MDS/MPN nature of the disease. The ICC retains the name a aCML for this entity. The ICC has additional criteria for cytopenia and eosinophils < 10%, which may result in different diagnoses by the two classifications. Furthermore, the ICC adds *SETBP1* and *ASXL1* mutations as molecular supportive criteria and the WHO-HAEM5 adds *SETBP1* and *ETNK1* mutations.

The ICC adds a provisional entity called MDS/MPN with isolated isochromosome 17q. However, whether this is a distinct entity or falls within the spectrum of aCML, with which it shares a similar genomic signature, is still being determined.

Myelodysplastic syndrome and myeloproliferative neoplasm with ring sideroblasts (RS) and thrombocytosis are renamed MDS/MPN with *SF3B1* mutation and thrombocytosis, in the presence of an *SF3B1* mutation of any VAF (WHO-HAEM5) or VAF ≥ 10% (ICC). In cases with wild type *SF3B1*, RS replaces *SF3B1* in the name in the WHO-HAEM5, while the ICC keeps *SF3B1* in the name and adds the qualifier NOS.

### Myelodysplastic neoplasms or syndromes

The WHO-HAEM5 has changed the name of myelodysplastic syndromes to myelodysplastic neoplasms while retaining the abbreviation MDS, to emphasise the neoplastic nature of MDS and harmonise the terminology with MPN.

Both classifications have undergone significant restructuring. Although there are many discrepancies in the grouping and nomenclature of MDS subtypes, the two classifications largely agree in terms of grouping based on genetic risk stratification. In the WHO-HAEM5, the number of dysplastic lineages is considered optional as these are usually dynamic and represent the phenotypic manifestation of clonal evolution rather than defining a specific MDS type. The ICC recognises that genetic risk stratification supersedes the influence of single-lineage dysplasia (SLD) versus multilineage dysplasia (MLD) on the prognosis of lower-risk MDS, but the number of dysplastic lineages is retained as subtypes of MDS, NOS. This is justified by studies showing inferior prognosis and distinct genetic profiles with MDS-MLD compared to MDS-SLD cases.^11^

The WHO-HAEM5 changes MDS with excess blasts (MDS-EB) to MDS with increased blasts (MDS-IB) while retaining long-standing thresholds for blast percentages. The ICC retains the term MDS-EB defined by the presence of 5% – 9% BM blasts or 2% – 9% PB blasts. Furthermore, the presence of EB supersedes any subtypes, except MDS with mutated *TP53*. The ICC introduces a novel category, MDS and AML (MDS/AML), corresponding to the WHO-HAEM5 category MDS-IB2, recognising the continuum between MDS and AML and the efficacy of novel therapeutic approaches that have been demonstrated in these patients.^12^ This was considered by the WHO-HAEM5 but not included because of subjectivity and the lack of a gold standard for blast counting.

The WHO-HAEM5 retains the 20% blast threshold for AML to prevent overtreatment that may arise with a lower threshold (e.g. 10%) while agreeing that MDS-IB2 can be considered AML-equivalent for therapeutic considerations and clinical trials.

Myelodysplastic syndrome with *TP53* mutation is added in both classifications, recognising the poor prognosis associated with *TP53* mutations.^13^ This subtype may be considered equivalent to AML for therapeutic considerations.^14^ Both classifications require ≥ 2 *TP53* gene disruptions or a single *TP53* mutation with VAF > 50%. The response to therapy of patients with a monoallelic *TP53* mutation is similar to those with wild-type *TP53* unless the *TP53* mutational allele burden is high.^15^

Myelodysplastic syndrome with mutated *TP53* is considered together with MDS/AML and AML with mutated *TP53* in the ICC as myeloid neoplasms with mutated *TP53* because of their overall similar aggressive behaviour that warrants a more unified treatment strategy across the blast spectrum.

The MDS-RS subcategories are changed in the WHO-HAEM5 to MDS with low blasts and *SF3B1* mutation (MDS-*SF3B1*) in patients with *SF3B1* mutations, while MDS with low blasts and RS is acceptable when there are ≥ 15% RS and wild type *SF3B1*. A similar change is seen in the ICC where neither dysplasia nor RS are required for the diagnosis of MDS-*SF3B1*.

However, in contrast to the WHO-HAEM5, those with WT *SF3B1* are classified as MDS, NOS.

The WHO-HAEM5 lists hypoplastic MDS (MDS-h) and MDS with fibrosis as new entities, while these are not classified separately by the ICC. Recognition of MDS-h is important as these patients may benefit from anti-thymocyte globulin therapy because of the immune-mediated pathogenesis of the disease being similar to aplastic anaemia.

Childhood MDS with low blasts (cMDS-LB) and childhood MDS with increased blasts (cMDS-IB) replace the term refractory cytopenias of childhood (RCC) in the WHO-HAEM5 but not the ICC. The ICC states that it is apparent that not all RCCs are true MDS and the pathogenesis remains incompletely understood.

### Myelodysplastic syndrome progression to myelodysplastic and myeloproliferative neoplasm

When MDS and MPN progress, the WHO-HAEM5 and ICC have substantial differences in the terminology. For instance, MDS-*SF3B1* that acquires a *JAK2* mutation with resulting thrombocytosis is re-classified as MDS/MPN-*SF3B1* according to the WHO-HAEM5, while it remains MDS-*SF3B1* in the ICC. Additionally, in the WHO-HAEM5, MDS that develops persistent proliferative features is re-classified as MDS/MPN-NOS, and MDS that develops monocytosis is re-classified as CMML. According to the ICC, MDS that later develops leucocytosis, monocytosis or thrombocytosis is called MDS with neutrophilic, monocytic or thrombocytic progression.^3^

### Systemic mastocytosis

The major criteria for systemic mastocytosis (SM) remain unchanged in the WHO-HAEM5 but are modified in the ICC, with the addition of tryptase and CD117 immunoreactivity detection to ensure correct identification of mast cells. Any *KIT* mutation causing ligand-independent activation is sufficient for the minor criterion. In addition, CD30 expression is added to the immunophenotype minor criterion. These changes are made to recognise well-differentiated SM (WDSM).^16^ Importantly, SM with an associated haematologic neoplasm (SM-AHN) is retained by the WHO-HAEM5 while it is restricted in the ICC to SM with an associated myeloid neoplasm (SM-AMN). The latter is supported by data showing that genetic mutations are not shared in SM and lymphoid neoplasms, but rather the conditions occur concomitantly.^17^

### Acute myeloid leukaemia

The classification of AML is restructured to emphasise major breakthroughs made over the past few years. The WHO-HAEM5 categorises AML into AML with defining genetic abnormalities and AML defined by differentiation thus phasing out AML, NOS. The ICC lists all AML subtypes without subcategorisation and retains the previous name AML, NOS for those AMLs without defining genetic abnormalities.^2^ Both classifications expand the *KMT2A* and *MECOM* rearrangement categories to allow for many fusion partners, and the ICC includes defined partner genes to provide a more genetically defined classification.^3^ Both classifications omit mutated *RUNX1* as a separate AML entity, as *RUNX1* mutations are not specific enough to represent a distinct type of AML. The ICC adds *RUNX1* mutations as defining for AML with myelodysplasia-related (MR) gene mutations.

While both classifications retain the ≥ 20% blast cut-off for AML, NOS (ICC) and AML defined by differentiation (WHO-HAEM5), the blast count for AML with defining genetic abnormalities differs between the two classifications. The ICC requires ≥ 10% blasts for most AML categories with defining genetics other than for *BCR::ABL1* and mutated *TP53*, where ≥ 20% blasts distinguish it from CML, and for AML-MR where ≥ 20% blasts distinguish it from MDS. The WHO-HAEM5 omits the blast count for AML with defining genetics except for *CEBPA* mutations, *BCR::ABL1* and AML-MR. The WHO-HAEM5 emphasises the correlation of morphological and molecular findings, including the clone size (VAF) of the defining genetic alteration, to ensure that the genetic abnormality is the driver mutation of disease pathology. The implication is that patients with low blast counts, even ≤ 5%, will require molecular testing to exclude AML-defining genetic abnormalities, adding to the economic burden of diagnosis.

Regarding *CEBPA* mutations, the ICC considers only bZIP mutations stating that these are prognostically informative and require ≥ 10% blasts, while the WHO-HAEM5 additionally allows biallelic mutations (*biCEPBA*) and requires ≥ 20% blasts.^18^

Another clinically relevant difference is the inclusion of a novel entity of MDS/AML in the ICC, but not the WHO-HAEM5, defined by the presence of 10% – 19% blasts in the BM and *TP53* mutation or MR abnormality. The ICC also defines AML with mutated *TP53* (≥ 20% PB or BM blasts) as a distinctly aggressive AML, whether presenting *de novo*, as progression of MDS or as therapy-related disease, because of the dismal prognosis regardless of morphological variant.^3,19^ Any somatic *TP53* mutation with VAF > 10% is sufficient for this diagnosis. The majority of pure erythroid leukaemias will be classified here as they often have *TP53* mutations. The WHO-HAEM5 does not recognise this as a distinct AML entity but does consider *TP53* in MDS.

Both classifications agree on many of the changes made to the AML-MR category, although there are differences in nomenclature and classification. Importantly, the morphological-driven diagnostic criteria (SLD and MLD) are omitted and the defining cytogenetic abnormalities are expanded. The only difference in defining characteristics between classifications is the inclusion of *RUNX1* mutations by the ICC. Both classifications agree that MLD is a proxy for underlying poor prognostic MR genetic abnormalities, and that molecular definition is preferable to distinguish AML-MR from other entities associated with dysplasia such as AML with *NPM1* or *biCEPBA*.

Both classifications introduce a section allowing genetic aberrations that are not currently listed as part of the classification, as these could possibly become defined entities in future editions. This is named AML with other defined genetic alterations, where any blast count is acceptable, in the WHO-HAEM5 and AML with other rare recurrent translocations, where ≥ 10% blasts are required, in the ICC.

Overall, the differences between the classification systems may lead to different diagnoses depending on the system used, especially at blast counts of ≤ 10%. This will have a significant impact on clinical decision-making, clinical trial design, conduct and interpretation, and regulatory aspects of therapies, disease registries and health system administration such as drug reimbursements.^20^

### Secondary myeloid neoplasms and myeloid neoplasms with germline predisposition

The WHO-HAEM5 groups all myeloid neoplasms that arise secondary to exposure to cytotoxic therapy or germline predisposition in this category of secondary myeloid neoplasms. Acute myeloid leukaemia transformation of MPN is retained in the MPN category, and AML transformation of MDS and MDS/MPN remains in AML-MR. The ICC identifies these associations, that is therapy-related, germline predisposition and progressing from MDS and MDS/MPN, as diagnostic qualifiers rather than as specific disease categories. Thus, the ICC does not have a stand-alone category for therapy-related secondary myeloid neoplasms.

The ICC has a separate category for paediatric and/or germline mutation-associated disorders. Any underlying germline predisposition mutation or syndrome should be specified as a qualifier after the MDS, AML or other malignancy diagnosis and subtype. The WHO-HAEM5 restructures this entity to include myeloid neoplasms associated with germline predisposition (MNGP) that were previously classified elsewhere, such as MNGP associated with inherited bone failure syndromes and telomere biology disorders as well as myeloid proliferations associated with Down syndrome, both of which are contained in the MNGP and potential organ dysfunction category. Similarly, the ICC classifies these disorders in the category of haematological neoplasms with germline predisposition (HNGP) associated with a constitutional disorder affecting multiple organ systems. New rare germline subtypes are added into both classifications.

### Myeloid or lymphoid neoplasms with eosinophilia and gene rearrangement

The defining gene rearrangements are expanded to include *JAK2* rearrangement, *FLT3* rearrangements, *ETV6::ABL1* fusion and, in the WHO-HAEM5, other rarer defined tyrosine kinase fusions.

### Acute leukaemia of ambiguous lineage

Both classifications include acute undifferentiated leukaemia (AUL) and mixed phenotype acute leukaemia (MPAL) in the acute leukaemia of ambiguous lineage (ALAL) category. In addition, MPALs are subtyped according to genetic findings (*BCR::ABL1* and *KMT2A* rearrangement) or by immunophenotype (B-cell, T-cell or myeloid). Lineage assignment is revised in the WHO-HAEM5 to emphasise that the intensity of lineage antigen expression should be similar to the normal population.

The WHO-HAEM5 defines two new subtypes, MPAL with *ZNF384* rearrangement and ALAL with *BCL11B* rearrangement. The ICC does not include these in the ALAL category. However, the ICC mentions, in B-lymphoblastic leukaemia or lymphoma (B-ALL) with *ZNF384* rearrangement, that expression of myeloid antigens is common and may be sufficient for a diagnosis of B-cell and myeloid (B/myeloid) MPAL. *BCL11B* rearrangement is observed in AUL, T-cell and myeloid (T/myeloid) MPAL, early T-precursor lymphoblastic leukaemia (ETP-ALL) and AML.^21^ The ICC includes *BCL11B* rearrangement only as a recurrent genetic abnormality in ETP-ALL. While *BCL11B* rearrangement may be detected on karyotype, *ZNF384* rearrangements require fluorescence in situ hybridisation (FISH), reverse transcription-polymerase chain reaction (RT-PCR) or NGS.^22^ Diagnostic testing for this specific variant is currently not available as part of the routine testing repertoire in South Africa.

### Histiocytic and dendritic cell neoplasms

In the ICC, this category follows the lymphoid neoplasms while in the WHO-HAEM5, it follows myeloid neoplasms in accordance with ontogeny. The WHO-HAEM5 designates four subtypes, namely plasmacytoid dendritic cell (DC) neoplasms, Langerhans cell neoplasms, other DC neoplasms and histiocytic neoplasms. New to this category are the plasmacytoid DC neoplasms, which consist of mature plasmacytoid DC proliferations associated with myeloid neoplasms and the blastic plasmacytoid DC neoplasms, the latter classified as its own category in the ICC.

Both classifications add two new subtypes, namely ALK-positive histiocytosis and Rosai-Dorfman-Destombes disease. Rosai-Dorfman-Destombes disease can be a benign condition but in the presence of MAPK pathway gene mutations, it is considered a histiocytic neoplasm.^23^

The WHO-HAEM5, but not the ICC, omit two subtypes from this category, namely follicular DC sarcoma and fibroblastic reticular cell tumour. There are minor name changes in the ICC, favouring terms such as histiocytosis or sarcoma over tumour.

While there are some differences in the categorisation of neoplasms between the WHO-HAEM5 and ICC and minor differences in the naming of entities, the diagnostic criteria are similar.

## Lymphoid neoplasms

### Precursor lymphoid neoplasms

B-lymphoblastic leukaemia or lymphoma and T-lymphoblastic leukaemia or lymphoma (T-ALL) are in separate categories in both classifications.

The ICC includes a subclassification for *BCR::ABL1*-positive B-ALL to distinguish sole lymphoid involvement from multilineage involvement. For the latter, FISH evidence of *BCR*::*ABL1* in granulocytes as well as lymphoblasts is required as there is evidence that these entities may require different management.^24^ Both classifications include B-ALL with *BCR-ABL1*-like features as an entity (previously provisional) with promising targeted therapy response in preclinical models.^25^ The ICC further divides this category into *ABL-1* class rearranged, *JAK-STAT* activated and NOS, as the *ABL-1* class responds better to TKI therapy.^26^

Both classifications introduce new entities defined by genetic abnormalities, more so in the ICC than in the WHO-HAEM5. The ICC also adds provisional entities. Of note, B-ALL with *ZNF384* or *ZNF362* rearrangement is added in the ICC. In addition to B-ALL, the rearrangement is seen in 50% of B/myeloid MPAL in children, but not in adults. The *ZNF384* rearrangement is not included in B-ALL in the WHO-HAEM5 but is included in MPAL.

The WHO-HAEM5 clarifies the good prognostic group of B-ALL with high hyperdiploidy, defined by 51–65 chromosomes. The WHO-HAEM5 describes three subtypes of B-ALL with hypodiploidy, namely high-hypodiploid (40–43 chromosome), low hypodiploid (32–39 chromosome) and near haploid (24–31 chromosome). The ICC recognises only the latter two entities, although the prognosis is poor across hypodiploid subtypes.

### Mature B-cell neoplasms

The WHO-HAEM5 uses a hierarchical taxonomy, which allows for less specific diagnosis when the biopsy is suboptimal or has limited material, or molecular and/or genetic testing is not available in limited resource countries or centres. This hierarchical taxonomy makes the WHO-HAEM5 classification globally applicable. The ICC lists the indolent entities first, then the aggressive entities. The ICC places emphasis on the role of genomic profiling in the diagnosis of mature lymphoid neoplasms. The WHO-HAEM5 implements essential and desirable diagnostic criteria, which encourages the use of molecular testing when essential for diagnosis and enables diagnosis in the absence of molecular results when desirable.

Mantle cell leukaemia remains unchanged in both classifications. The WHO-HAEM5 includes new umbrella groups, transformation of indolent B-cell lymphomas (not part of the ICC) and lymphoid proliferations and lymphomas associated with immune deficiency and dysregulation. A new category, tumour-like lesions with B-cell predominance (not part of the ICC) is added in the WHO-HAEM5, which includes reactive B-cell rich lymphoid proliferations that can mimic lymphoma, IgG4-related disease, unicentric Castleman disease, idiopathic multicentric Castleman disease and Kaposi sarcoma herpesvirus or human herpesvirus 8 (KSHV/HHV8)-associated multicentric Castleman disease. Recognising the proven origin of Hodgkin lymphoma (HL) from B-cells, this is included in the B-cell lymphoid proliferations and lymphomas category in the WHO-HAEM5 but remains a separate category in the ICC.

### Small lymphocytic proliferations

The diagnostic criteria for chronic lymphocytic leukaemia (CLL) remain unchanged. Prolymphocytic progression of CLL is a new term introduced in the WHO-HAEM5.

The definition of monoclonal B-cell lymphocytosis (MBL) remains unchanged in the ICC. The WHO-HAEM5 omits the atypical CLL type and adds low-count MBL or clonal B-cell expansion for clonal CLL phenotype B-cells < 0.5 × 10^9^/L.

### Splenic B-cell lymphomas and leukaemias, and B-cell prolymphocytic leukaemia

Splenic B-cell lymphomas and leukaemias is a new category in the WHO-HAEM5, which groups all the B-cell lymphomas and leukaemias that are characterised by splenomegaly in the absence of prominent lymphadenopathy. These entities include hairy cell leukaemia (HCL), splenic marginal zone lymphoma and leukaemia (SMZL), splenic diffuse red pulp small B-cell lymphoma and leukaemia (SDRPL) and splenic B-cell lymphoma and leukaemia with prominent nucleoli (SBLPN). Splenic diffuse red pulp small B-cell lymphoma and leukaemia is considered an entity in the WHO-HAEM5, while it remains a provisional entity in the ICC.

B-cell prolymphocytic leukaemia (B-PLL) is omitted in the WHO-HAEM5 as it can be better classified as SBLPN, leukaemic mantle cell lymphoma (MCL) or prolymphocytic progression of CLL. Splenic B-cell lymphoma and leukaemia with prominent nucleoli includes previously named entities B-PLL, HCL-variant (HCL-v) and some SMZL. The ICC does not recognise SBLPN. They retain B-PLL and recommend making the diagnosis of B-PLL in *de novo* cases. They also retain HCL-v as a provisional entity. Both HCL-v and SDRPL are provisionally grouped in the category of unclassifiable splenic B-cell lymphomas in the ICC. Different naming conventions between the classifications may lead to confusion and complicate management in these entities.

### Lymphoproliferative neoplasms characterised by immunoglobulin M

The diagnostic criteria of immunoglobulin M (IgM) monoclonal gammopathy of undetermined significance (MGUS) and lymphoplasmacytic lymphoma (LPL) or Waldenström macroglobulinaemia (WM) are similar in the two classifications. The ICC introduces two IgM MGUS entities, namely plasma cell type and NOS (Table 1).^27^ The WHO-HAEM5 introduces two subtypes of LPL, namely IgM-LPL type (95% of cases) and non-LPL type (5% of cases). The latter include cases with monoclonal immunoglobulin G (IgG) or immunoglobulin A (IgA) monoclonal proteins, non-secretory LPL and IgM LPL without BM involvement.

**TABLE 1:** Features to differentiate IgM monoclonal gammopathy of undetermined significance, lymphoplasmacytic lymphoma or Waldenström macroglobulinemia and cold agglutinin disease.^28,29^

Monoclonal immunoglobulins	IgM MGUS		LPL or WM		CAD (IgM [91%] > IgG [4.5%] > IgM + IgG [2.8%])
	Plasma cell type (IgM)	NOS (IgM)	IgM	Non-IgM (IgG or IgA)	
Clonal plasma cells	< 10%	< 10%	> 10%	> 10%	Yes
Monoclonal B-cells	No	Yes	Yes	Yes	Yes
Lymphoplasmacytic B-cell aggregates in the trephine biopsy	Absent	Absent	ICC: Present WHO-HAEM5: > 10%	ICC: Present WHO-HAEM5: > 10%	Present
*MYD88* L265P mutation	Absent	Present	Present or absent	Present or absent	Absent
Other	The t(11;14) *IGH::CCND1* or other myeloma-associated IGH rearrangements may be present	May transform to LPL	*CXCR4* mutations in 40%. Associated with symptomatic hyperviscosity and resistance to ibrutinib^8,9^	-	Trisomies of chromosomes 3, 12 and 18. *KMT2D* and *CARD11* mutations

IgM MGUS, IgM monoclonal gammopathy of undetermined significance; LPL or WM, lymphoplasmacytic lymphoma or Waldenström macroglobulinemia; NOS, not otherwise specified; CAD, primary cold agglutinin disease; WHO-HAEM5, haematolymphoid tumours from the revised 5th edition; ICC, International Consensus Classification; IgM, immunoglobulin M; IgG, immunoglobulin G; IgA, immunoglobulin A.

Primary cold agglutinin disease (CAD) is a new entity included in both classifications as an entity distinct from LPL and IgM MGUS, with IgM-related symptoms, but no tumour-related symptoms (Table 1).

### Extranodal marginal zone lymphoma and nodal marginal zone lymphoma

The diagnostic criteria for extranodal and nodal marginal zone lymphoma (MZL) remain unchanged. Primary cutaneous MZL is a new entity in the WHO-HAEM5, with the ICC equivalent entity being primary cutaneous MZ lymphoproliferative disorder (LPD). Paediatric nodal MZL (PNMZL) is a distinct entity in the WHO-HAEM5.

Immunohistochemistry for immune receptor translocation-associated protein 1 (IRTA1) and myeloid nuclear differentiation antigen (MNDA) may aid in diagnosis by distinguishing mucosa-associated lymphoid tissue (MALT) gastric lymphoma from chronic gastritis or reactive lymphocyte hyperplasia, and is also used to exclude non-cutaneous primary disease from PNMZL.^30^ However, these immunohistochemical stains are not available in South Africa.

### Follicular lymphoma

The WHO-HAEM5 introduces three morphological subtypes of follicular lymphoma (FL), namely classic FL (cFL) consisting of follicular growth of centrocytes and centroblasts, and t(14;18)(q32;q21) associated with *IGH::BCL2* fusion; follicular large B-cell lymphoma (FLBL) consisting of a follicular pattern with an area of diffuse large B-cell lymphoma, and rarely carries the *BCL2* translocation; and FL with uncommon features (uFL) consisting of FL with blastoid or large centrocyte features (high proliferation index and MUM1 or IRF4 expression) and FL with predominantly diffuse growth pattern (also known as diffuse follicular lymphoma variant) associated with CD23 expression, an absence of *IGH::BCL2* fusion and frequent *STAT6* mutations along with *1p36* deletion or *TNFRSF14* mutation.^31^

The WHO-HAEM5 phases out the grading system for FL, justified by the lack of reproducibility and clinical significance in the era of modern therapy. The ICC retains the grading system but acknowledges that its significance is debatable. Both classifications recognise that grade 3B should be treated as diffuse large B-cell lymphoma (DLBCL). The ICC recognises a new distinct entity, namely testicular FL and a provisional entity called BCL2-R-negative, CD23-positive follicular centre lymphoma. The latter correlates with diffuse follicular lymphoma variant, which further supports the argument that a single classification system should be developed.

### Transformations of indolent B-cell lymphomas

The WHO-HAEM5 defines this entity as the transformation of an indolent B-cell lymphoma, such as CLL, FL or MZL, to an aggressive large B-cell lymphoma (LBCL). It includes Richter’s transformation of CLL.

### Large B-cell lymphoma

Both classifications retain the cell-of-origin subtyping for DLBCL, NOS as immunohistochemistry is widely used, simple, low cost and has potential prognostic impact. However, gene expression profiles remain the gold standard, which can explain the biological complexity.

In the ICC, primary DLBCL of the testis is considered a distinct entity, while the WHO-HAEM5 introduces LBCL of immune-privileged sites for LBCL occurring in immune sanctuaries such as the blood–brain, blood–retina and blood–testis barriers.^32^

Large B-cell lymphoma with IRF4 rearrangement is a distinct entity in both classifications. Burkitt-like lymphoma with 11q aberration is renamed LBCL with 11q aberration in the ICC and high-grade B-cell lymphoma (HGBCL) with 11q aberration in the WHO-HAEM5. This is based on molecular studies showing similarity to DLBCL rather than Burkitt lymphoma (BL), for example, lacks MYC-rearrangement but harbours an 11q gain/loss pattern.^33^

Fibrin-associated LBCL is a distinct entity in the WHO-HAEM5, while in the ICC it is still considered a subtype of DLBCL associated with chronic inflammation. The WHO-HAEM5 introduces the new entity, fluid overload-associated LBCL, while the ICC introduces the new provisional entity, HHV8 and Epstein–Barr virus (EBV)-negative primary effusion-based lymphoma.

### High-grade B-cell lymphomas

The ICC classifies double-hit (DH) HGBCL into two entities, namely HGBCL with *MYC* and *BCL2* rearrangements (HGBCL-DH-BCL2) (with or without *BCL6* rearrangement) and a provisional entity of HGBCL with *MYC* and *BCL6* rearrangements (HGBCL-DH-BCL6). The WHO-HAEM5 specifies that DH lymphomas require *MYC* and *BCL2* rearrangements, called DLBCL or HGBCL with *MYC* and *BCL2* rearrangements because those with *BCL6* rearrangement have a diverse gene expression signature. Those with *BCL6* rearrangement are considered a genetic subtype of either DLBCL, NOS and HGBCL, NOS in the WHO-HAEM5. Both classifications consider terminal deoxynucleotidyl transferase (TdT) expression in the absence of CD34 as DLBCL or HGBCL, NOS with expression of TdT rather than B-ALL.^34,35^

### Burkitt lymphoma

The WHO-HAEM5 highlights two subtypes of BL, namely EBV-positive BL and EBV-negative BL, to reflect the viral rather than mutational mechanism of BL pathogenesis. Neoplasms with a precursor B-cell phenotype and *MYC* rearrangement are referred to as B-ALL with *MYC* rearrangement in the ICC.

### Large B-cell lymphoproliferative disorders related to viral agents

Epstein–Barr virus-positive polymorphic B-cell LPD, NOS (EBV-PBLPD, NOS) is a new entity in the ICC. This entity is reserved for cases with a change in lymph node architecture because of an EBV-positive polymorphic infiltrate that does not meet the criteria for a diagnosis of lymphoma, such as EBV-positive DLBCL, NOS and EBV-positive classic HL (cHL). Epstein–Barr virus-positive mucocutaneous ulcers (EBV-MCU) is a separate entity in the ICC, associated with a good prognosis.^36,37^ Conversely, the WHO-HAEM5 include EBV-MCU and EBV-PBLPD, NOS in the lymphoid proliferations and lymphoma associated with immune deficiency and dysregulation category.

### Lymphoid proliferations and lymphomas associated with immune deficiency and dysregulation

This is a category introduced in the WHO-HAEM5. It is based on a three-part approach: firstly, histological diagnosis; secondly, presence or absence of a virus (EBV, HHV8); and thirdly, clinical setting and immune deficiency background (post-transplant, HIV, iatrogenic and autoimmune diseases, inborn error of immunity).

### Hodgkin lymphoma and nodular lymphocyte predominant Hodgkin or B-cell lymphoma

The WHO-HAEM5 retains nodular lymphocyte predominant HL (NLPHL) in the HL category and moves it to the B-cell lymphoma category. The ICC retains cHL in its own category. Additionally, the name of NLPHL is changed to nodular lymphocyte predominant B-cell lymphoma and is categorised in the mature B-cell neoplasms, based on the key biological and clinical differences from cHL.^38,39^

### Mediastinal grey zone lymphoma

The category, B-cell lymphoma unclassifiable with features intermediate between DLBCL and cHL is replaced by mediastinal grey zone lymphoma (MGZL) in both classifications. This is supported by growing evidence that MGZL represents a biological continuum with cHL and primary mediastinal B-cell lymphoma (PMBL) rather than morphological mimics.^40,41^

### Plasma cell neoplasms

The WHO-HAEM5 includes monoclonal gammopathy of renal or clinical significance (MGRS or MGCS) as an entity, while the ICC considers it a clinical feature to be added to the diagnosis of non-IgM MGUS. The ICC recognises two types of IgM MGUS, namely plasma cell type and NOS type. IgM MGUS, NOS may show *MYD88* mutation or monoclonal B-cells and a lack evidence of other small B-cell neoplasms.^42,43^

The ICC introduces four multiple myeloma (MM) entities with recurrent cytogenetic abnormalities, namely MM with *CCND* family translocations, MM with *NSD2* translocation, MM with *MAF* family translocations and MM with hyperdiploidy. Finally, MM without cytogenetic abnormalities is referred to as MM, NOS.^44,45^ Interphase FISH is recognised as the technique of choice for cytogenetic characterisation, with published consensus FISH panels for MM.^46^

There are no changes in the definition of smouldering (asymptomatic) myeloma. Minimal bone marrow involvement detected by flow cytometry is of prognostic importance for solitary plasmacytomas of bone, therefore clonal plasma cells of 10% should form part of the diagnosis of these entities.^47^ Primary amyloidosis is renamed immunoglobulin-related (AL) amyloidosis in the WHO-HAEM5 and Ig light chain (AL) amyloidosis in the ICC, distinguishing the systemic from the localised form.

Among plasma cell neoplasms with a paraneoplastic syndrome where the diagnosis is based on clinical and imaging studies, the syndromes POEMS (polyneuropathy, organomegaly, endocrinopathy, monoclonal gammopathy and skin changes) and TEMPI (telangiectasia, erythrocytosis with elevated erythropoietin level, monoclonal gammopathy, perinephric fluid collection and intrapulmonary shunting) are defined entities in both classifications. The WHO-HAEM5 introduces a new entity, AESOP syndrome (adenopathy and an extensive skin patch overlying a plasmacytoma).^48^

### T-cell and natural killer-cell lymphoid proliferations and lymphomas

Tumour-like lesions with T-cell predominance is a new category in the WHO-HAEM5 comprising of three entities that are not malignant but have a proliferation of T-cells that can mimic T-cell lymphoma and lead to misdiagnosis.^49^

T-lymphoblastic leukaemia or lymphoma is in its own category, separate from B-ALL, in both classifications. Early T-cell precursor ALL with *BCL11B* rearrangement is a new subtype in the ICC. Both classifications modify the subtype T-ALL with the addition of NOS if it does not meet the criteria for other subtypes.^26,49^

Natural killer (NK)-lymphoblastic leukaemia or lymphoma, a previous provisional entity, is omitted in the WHO-HAEM5 because of a lack of evidence of clear diagnostic criteria and significant overlap with other entities.^49,50^

Natural killer-large granular lymphocytic leukaemia is the new name in the WHO-HAEM5, which replaces chronic LPD of NK cells because of many similarities with T-large granular lymphocytic leukaemia.^49^

Primary cutaneous T-cell lymphoid proliferations and lymphomas is a new category in the WHO-HAEM5, which includes nine entities that better classify all primary cutaneous T-cell lymphomas that were previously separated in the WHO-HAEM4R. A change in nomenclature from lymphoma in primary cutaneous acral CD8-positive T-cell lymphoma to LPD was made in both classifications. A new entity called primary cutaneous peripheral T-cell lymphoma, NOS is added to this category in the WHO-HAEM5, which includes cases that cannot be classified under the other eight entities.^27,49,51^

Intestinal T-cell and NK-cell lymphoid proliferations and lymphomas include a name change from the former indolent T-cell LPD of the gastrointestinal tract. The WHO-HAEM5 changes the name to indolent T-cell lymphoma of the gastrointestinal tract because of the proven disease dissemination and significant morbidity. The ICC changes the name to indolent clonal T-cell LPD of the gastrointestinal tract to emphasise the monoclonal nature of the disease. Indolent NK-cell LPD of the gastrointestinal tract is a new entity added to this category in both classifications. This disease, previously thought to be reactive, has been shown to be neoplastic but with a benign clinical course. The ICC adds refractory celiac disease type II as a precursor to the entity, enteropathy-associated T-cell lymphoma.^27,49,52^

Hepatosplenic T-cell lymphoma has been reviewed in the WHO-HAEM5 in terms of epidemiology and morphology. A recent study has shown that this disease occurs in about half of patients > 60 years old and≈that BM dysplasia may be present, which can be confused with MDS.^49,53,54^

The WHO-HAEM5 introduces new nomenclature for the nodal T-follicular helper (TFH) cell lymphomas category, comprising of three renamed entities. The name changes better highlight the shared phenotypes and genotypes of the diseases.^55,56^ The ICC has similar changes, omitting the word nodal with the same three subtypes as in the WHO-HAEM5. Both classifications recognise the value of genetic profiling in the diagnosis of nodal TFH lymphoma because of its unique mutational landscape. These entities are considered to be part of one spectrum and patients may switch between patterns over time. Clinical management is not impacted by these subtypes.^27,49^

Among the EBV-positive NK or T-cell (NK/T-cell) lymphomas, the WHO-HAEM5 omits the term nasal-type from extranodal NK/T-cell lymphomas because this disease also occurs at other extranodal sites. Nodal EBV-positive T- and NK-cell lymphoma, previously listed as a variant of peripheral T-cell lymphoma, NOS, is a distinct entity in both classifications because of typical phenotypic and genetic findings.^27,49,57^

Both classifications introduce revised terminology for EBV-positive T- and NK-cell lymphoid proliferations and lymphomas of childhood, which better highlight the clinical and pathological features of these disorders.^27,49^

### Genetic predisposition syndromes

The WHO-HAEM5 includes new chapters on genetic predisposition syndromes associated with haematological tumours with two particular conditions highlighted, that is ataxia telangiectasia and Nijmegen-breakage syndrome. These chapters include recommendations for germline mutation testing and emphasise the importance of diagnosis for treatment, monitoring and counselling of families.^49^

### Stroma-derived neoplasms of lymphoid tissues

This is a new category in the WHO-HAEM5 that includes new, revised and recategorised tumours. The new entities include mesenchymal neoplasms, particularly those of the lymph nodes and spleen. The EBV-positive inflammatory follicular DC sarcoma is a revised separate entity with a change in nomenclature because of its specific clinical and pathological aspects.^58^ The ICC prefers the term tumour to sarcoma because of the indolent nature of this disease.^27^ Follicular DC sarcoma and fibroblastic reticular cell tumours, which were previously categorised under histiocytic and DC neoplasms, have been moved to this new category because of their mesenchymal origin.^49,59^

## Conclusion

There have been huge strides in the classification of haematolymphoid tumours with regard to morphologic, immunophenotypic, molecular and cytogenetic characterisation. It is also reassuring to have the stepwise approach to diagnosis allowing developing countries that do not have access to advanced genetic tests to still be able to make appropriate diagnoses using available testing methods.

Two classification systems are unnecessary and there needs to be harmonisation to allow for a universal diagnostic system. The WHO and ICC share many similarities with minor name changes and subgrouping. It is observed that the ICC is the only system that introduces provisional entities. There are a few instances where diagnostic grouping differs between the classifications which could have clinical implications regarding treatment and enrolment in clinical trials. This further poses the need for a shared classification system for the benefit of the patient. The authors acknowledge that both classification systems have merit and either could be adopted by individual institutions at present.

A major concern for the large number of nations with limited resources remains the specialised testing requirements for the definition of the various diagnostic entities and disease activity categories. While some of these testing modalities and methodologies may be standard of care in more affluent societies, they are simply not available to many lower- to middle-income countries.

There is universal agreement about the need for the science-grounded objective criteria for the classification of haematolymphoid tumours. The authors therefore appeal for interim advocacy measures to assure access to the specific diagnostic investigations in poorly resourced societies, pending more permanent and sustainable access. While the *quid pro quo* for such diagnostic testing equity may not be apparent initially, the long-term benefits for medical science and humanity, in general, are easy to hypothesise.
